# Clinical Study of 3DCRT Combined with SBRT in the Treatment of Patients with EGFR Mutation Oligometastatic Non-Small Cell Lung Cancer

**DOI:** 10.1155/2023/1266778

**Published:** 2023-02-16

**Authors:** Deng Wang, Yingbang Wan, Lihua Wang

**Affiliations:** ^1^Ezhou Central Hospital (Respiratory and Critical Care Unit), Ezhou, Hubei 436000, China; ^2^Department of Respiratory Endology, People's Hospital of Dongxihu District, Wuhan, Hubei 430040, China

## Abstract

**Background:**

Lung cancer is one of the malignant tumors with the highest morbidity and mortality in my country and the world. Among them, non-small-cell lung cancer (NSCLC) accounts for about 80%. For patients who are diagnosed with NSCLC and have epidermal growth factor receptor, EGFR gene-sensitive mutations The treatment is particularly important.

**Aims:**

To investigate the efficacy and prognosis of 3DCRT combined with local SBRT in patients with EGFR mutation oligometastatic NSCLC.

**Materials and Methods:**

Eighty patients with EGFR mutation oligometastatic NSCLC were selected by random remainder grouping method. 3DCRT combined with SBRT is effective and safer in patients with EGFR-mutant oligometastatic NSCLC, and significantly improves the patient's immune and tumor marker levels. It has a certain reference value in the clinical treatment of EGFR-mutant oligometastatic NSCLC.

## 1. Introduction

Lung cancer is the malignant tumor that causes the most deaths in the world. Most patients are already in stages III to IV when they see a doctor and cannot be removed by radical surgery [1]. For locally advanced nonresectable NSCLC, the main treatment currently recommended is still a combination of chemotherapy and radiotherapy. In particular, radiotherapy plays an extremely important role in the treatment of these patients [2]. Patients with peripheral locally advanced lung cancer, in addition to the primary lung tumors, are also accompanied by mediastinal lymph node metastasis. Therefore, the scope of radiotherapy covers mostly the primary lung and the area of mediastinal lymph node metastasis. The irradiation area is often larger. Great [3]. When formulating a radiotherapy plan, it is necessary to consider that normal organs are not exposed to excessively high doses, but also to ensure that the tumor receives a radical dose, which is a major problem in clinical practice [4]. Due to its special dosimetry advantages, the high dose of the irradiated target area, and the low dose of surrounding normal tissues, SBRT's status as a radical treatment in patients with early lung cancer has been confirmed by many studies, and it is recommended for the early stage of intolerable surgery. Patients with lung cancer [5].

Based on this, our department has explored the effect of 3DCRT combined with local SBRT consolidation therapy on the efficacy and prognosis of EGFR-mutant oligometastatic NSCLC. The current research results are reported as follows.

## 2. Material and Methods

### 2.1. Exclusion Criteria

Inclusion criteria were as follows: (i) diagnostic criteria for EGFR mutation oligometastatic NSCLC [6]; (ii) pathological diagnosis of NSCLC and positive EGFR driver gene mutation. The Eastern Cooperative Oncology Group (ECOG) score [7]: 0–2, acquired drug resistance after first and second-generation EGFR-TKI treatment and expected survival time ≥3 months; local treatments (such as surgical resection, radiotherapy, microwave ablation, radiofrequency ablation, seed implantation, argon, and helium knife) were received for the progressive lesions. The T790M gene mutation test result was negative before local treatment, and it reappeared after local treatment. The disease progresses and the treatment plan is changed.

Exclusion criteria were as follows: (i) patients whose EGFR-TKI resistance and the original EGFR-TKI combined with local treatment cannot be evaluated by imaging, and who have a recurrence of recurring cancer (except intraepithelial carcinoma) within 5 years; (ii) there are three types of patients, namely, pregnancy, ineffective contraception, and lactation.

### 2.2. Nursing Intervention Methods

The control group was given 3DCRT, namely.

#### 2.2.1. Radiotherapy Positioning

Lie on your back, put your arms around your elbows in front of your forehead, with the phantom fixed. After wearing the phantom, a C-enhanced scan was performed, with a thickness of 5 mm, and the scan range was from the hyoid bone to the lower pole of the kidney. SBRT is positioned three days before the end of 3DCRT, and the position is the same as that of 3DCRT. Lie on your back, put your arms around your elbows in front of your forehead, with the phantom fixed. CT scanning method: after the phantom is put on, an enhanced CT scan is performed, with a thickness of 5 mm, and the scanning range is from the hyoid bone to the lower pole of the kidney.

#### 2.2.2. Delineation of Tumor Target Areas and Organs at Risk

The target area of 3DCRT is delineated, GTV, and lung lesions are delineated when the chest CT window width is 1600HU and the window level is −600HU, and the mediastinal lesions are delineated when the chest CT window width is 400HU and the window level is 20HU. CTV, pulmonary lesions are GTV externally placed 6–8 mm; mediastinal lesions are the areas where metastatic lymph nodes are located, such as 4R area metastasis including the entire 4R area, or mediastinal lesions GTV externally placed 5 mm. PTV is CTV externally placed respiratory movement + positioning The error and respiratory movement are measured by the patient under a simulated positioning machine. The positioning error varies from 5 to 7 mm in each direction. The target area of SBRT is delineated, the lesion seen in the lung window by GTV, and the PTV is 0.5 cm of GTV. The delineation of organs at risk, including the spinal cord, normal lung tissue, trachea, esophagus, heart, and important nerves (such as the brachial plexus).

#### 2.2.3. Formulation of Radiotherapy Plan

3DCRT plan 3–5 field technology, requires a 95% isodose curve to surround 95% of PV, the divided dose is 2 Gy/time, 5 times/week, the total dose of 46–50 Gy, requires both lungs V20 < 25%, V30 < 18%, MLD < 18 Gy, and maximum spinal cord dose ≤40 Gy.

The treatment group implemented 3DCRT combined with SBRT, that is., the patient lies supine on a carbon fiber positioning bed, with both hands elbows lifted on top of the head, the position adopts a vacuum negative pressure bag fixing device, and the three-dimensional laser light determines the coordinates of the reference point and marks it on the skin. The marking point should be as close as possible to lesions. First, observe the movement trajectory of the tumor in the fluoroscopy mode of the simulated machine, and then in the state of free breathing, spiral-enhanced CT scans continuously with a layer spacing of 3mm. The scan range is from the entrance of the thorax to the bottom of the lung, including the whole lung and the lesion up and down 10–15 cm. The scan is completed then the data is transferred to the Philips Pinnacle9 treatment planning system workstation. After a senior physician with more than 7 years of radiotherapy experience performs the delineation and three-dimensional reconstruction of the target area and important organs, a physicist with more than 3 years of work experience sets up the field and makes a radiotherapy plan. Refer to the chest CT or PET-CT results before radiotherapy, and according to the definition requirements of ICRU No. 50 and No. 62 reports, the tumor lesions can be seen as gross tumor areas (GTV) in the treatment planning system according to the lung window imaging standards, and the GTV is expanded by 5mm to form a clinical target area (CTV), the planned target volume (PTV) is determined according to the range of tumor movement with breathing under fluoroscopy, positioning error, and the dose relationship of adjacent lesions. Generally, the PTV is formed by 3–5 mm of CTV. The target area was delineated in the Pinnacles9 radiotherapy planning system, using linear accelerator 6MV-X-rays with 6–12 coplanar fields and isocentric irradiation. The treatment plan was PTV45–60 Gy, divided by 5–10 times, and the bioequivalent dose (BED) was 70.5–120.0 Gy, once a day, 5 times a week. Use the dose volume histogram and isodose line chart of the treatment planning system to comprehensively evaluate all plans. The prescribed dose is required to meet more than 95% PV, and the 90% isodose line completely covers the target area. All planned dose limits for major blood vessels, large air ducts, ribs, spinal cord, chest wall, and other organs at risk refer to the United States Radio Oncology Cooperative Group (RTOG) No. 0236 agreement. Incoming the X-ray simulator control system to reset the patient, observe whether the irradiation field range meets the requirements of the treatment plan under fluoroscopy, otherwise reposition, and make a plan. If the plan is satisfactory, then perform calibration on the simulated CT to measure whether the distance from the tumor center to the front, back, left, and right body surfaces is consistent with the treatment plan system. When the reset and alignment are satisfied, the accelerator will be verified, and the electronic field imaging device will be verified at 0 degrees and 90 degrees, respectively. Radiotherapy is performed when the image matching error does not exceed 1mm, and the clinical requirements are fully met. Each treatment strict reset and position verification were carried out according to the above requirements before.

### 2.3. Observation Indicators

Beckman CytoFLEX flow cytometer detects CD4+, CD3+, and CD4+/CD8+. The curative effect judgment standard is based on the solid tumor curative effect evaluation standard RECIST to evaluate the short-term curative effect: the total diameter of the measurable target lesions increased by 20% and exceeded the minimum total observed (beyond the baseline, if the total decrease was not observed during treatment), and the minimum absolute value increased by 5 mm. Disease control rate (DCR) = (CR + PR + SD) the number of cases/total number of cases × 100%.

## 3. Results

### 3.1. Comparison of General Information

The general data of the two groups of patients, such as gender, average age, tumor diameter, and pathological type were not significantly different by *t*-test and chi-square test (*P* > 0.05). See Table 1.

## 4. Discussion

The dose of radiation therapy for locally advanced NSCLC is still being explored. As early as 40 years ago, Dr. Fletcher proposed that in radiotherapy, a dose of 50 Gy is required to eliminate subclinical lesions, a radiation dose of 60 Gy is required to eliminate small lesions visible on the image, and a radiation dose of 75 Gy is required to eliminate a 3 cm tumor. Larger tumors require 80 to 90 Gy of radiation therapy dose. Some retrospective and nonrandomized prospective research data also indicate that the higher the dose, the better the treatment effect. When treating NSCLC, increasing the dose may lead to a longer survival time. Therefore, how can we ensure that normal tissues are not affected by comparison? Under the premise of high-dose irradiation, increasing the dose of the tumor area as much as possible and improving the local control rate have become the main goals of radiotherapy for locally advanced NSCLC [8]. In clinical practice, many scholars have studied the application of SBRT in lung cancer. For NSCLC who cannot or are unwilling to undergo surgery due to medical diseases, compared with conventional radiotherapy, the local control rate of SBRT is significantly higher than that of conventional radiotherapy [9]. In the application of lung cancer, compared with conventional radiotherapy, SBRT is significantly higher than conventional radiotherapy in terms of local control rate for NSCLC who are unable or unwilling to undergo surgery due to medical diseases. It is for the above reasons that this study, aimed at patients with peripheral locally advanced NSCLC, first irradiated peripheral lung lesions and mediastinal lymph node area with 46–60 Gy using 3DCRT technology, and then repositioned, using SBRT radiotherapy for lung lesions to further increase the dose so that the equivalent biological dose of the tumor reaches 72 Gy–86 Gy, while the radiation dose to the lungs is significantly reduced [10].

In this study, two cases were selected for the comparison of the whole CRT plan and the 3DCRT + SBRT plan. It was found that the 3DCRT + SBRT irradiation method was used under the premise that the primary tumor received a radical dose (equivalent biological dose BED) of 66 Gy∼72 Gy. The V20, V30, and MLD of both lungs are lower than that of 3DCRT, which means that the probability of severe radiation pneumonia is reduced. Research reports in recent years have shown that radiation-induced lung injury is a dose-limiting factor for lung radiotherapy [11]. Under the premise of ensuring that the dangerous organs are not excessive, the BED of the primary tumor using the 3DCRT + SBRT irradiation mode is higher, which can reach 72Cy. In some cases with small lung lesions in this group, when the volume of B00ST is less than 50cm^3^, the lung BED of this lesion can reach about 80 Gy [12]. The following results we finally observed: (1) The use of 3DCRT + SBRT radiotherapy has fewer radiotherapy-related adverse reactions. Only one patient developed grade III acute radiation pneumonia. The cause was considered to be related to the induction of upper respiratory tract infection in the late stage of radiotherapy. In other patients, no serious radiation pneumonia was found, and no serious esophagus, heart, and tracheal reactions were found during radiotherapy [13]. (2) The local control rate is high. The local control rate within one year is 95.7%. The patients with recurrence in the field are due to excessive mediastinal lymph nodes. The dose of 3DCRT cannot control the mediastinal lymph nodes well, and because the BED of lung lesions is higher, no local recurrence was found [14].

Of course, there are still some shortcomings in this study. SBRT can only be applied to peripheral NSCLC. Due to the influence of normal organs such as heart, large blood vessels, and trachea, it is difficult to operate the peripheral tumor of mediastinal lymph nodes. The therapeutic dose can only be given by 3DCRT. Due to the presence of dangerous organs such as the spinal cord, the dose of mediastinal lymph nodes can only be given at a dose of about 60 Gy. Therefore, a small number of patients have the recurrent laryngeal nerve and superior vena cava compression due to mediastinal lymph node recurrence, and serious later symptoms affect the quality of life. Therefore, it is necessary to study a more reasonable dose of 3DCRT combined with SBRT for the control of mediastinal lesions.

## 5. Conclusion

In summary, 3DCRT combined with SBRT for patients with EGFR-mutant oligometastatic NSCLC has a better curative effect and is safer, and significantly improves the patient's immune and tumor marker levels.

## Figures and Tables

**Table 1:** Comparison of general information between the two groups $\left[n,\left(\bar{x},\pm s\right)\right]$.

Group	Gender (male/female)	Average age (age)	Tumor diameter (cm)	Pathological type		
				Squamous cell carcinoma	Adenocarcinoma	Squamous adenocarcinoma
Control group (40)	28/12	36.63 ± 8.32	13.31 ± 1.67	10	22	8
Treatment group (40)	29/11	36.62 ± 8.31	13.33 ± 1.25	11	23	6
*χ* ^2^/*t*	0.061	0.007	0.074	0.065	0.051	0.346
*P*	0.805	0.995	0.941	0.799	0.822	0.556

## Data Availability

The experimental data used to support the findings of this study are available from the corresponding author upon request.

## Additional Points

The implementation of a whole process informationized health management model combined with cardiac rehabilitation intervention for elderly patients with coronary heart disease after PCI can improve the quality of life and exercise endurance, and at the same time improve the patient's self-care ability. Design and peer review process.

## References

[1] Lee S. S., Cheah Y. K. (2019). The interplay between MicroRNAs and cellular components of tumour microenvironment (TME) on non-small-cell lung cancer (NSCLC) progression. *Journal of Immunology Research*.

[2] Imyanitov E. N., Iyevleva A. G., Levchenko E. V. (2021). Molecular testing and targeted therapy for non-small cell lung cancer: current status and perspectives. *Critical Reviews in Oncology*.

[3] Chae Y. K., Chang S., Ko T. (2018). Epithelial-mesenchymal transition (EMT) signature is inversely associated with T-cell infiltration in non-small cell lung cancer (NSCLC). *Scientific Reports*.

[4] Xie X., Zhang W., Wang H. (2021). Dynamic adaptive residual network for liver CT image segmentation. *Computers & Electrical Engineering*.

[5] Zeng K. L., Tseng C. L., Soliman H., Weiss Y., Sahgal A., Myrehaug S. (2019). Stereotactic body radiotherapy (SBRT) for oligometastatic spine metastases: an overview. *Frontiers Oncology*.

[6] Chinese Medical Association (2018). Chinese medical association Oncology branch, Chinese medical association journal press. Chinese medical association lung cancer clinical diagnosis and treatment guidelines (2018 edition). *Chinese Journal of Oncology*.

[7] Boran E., Ramantani G., Krayenbühl N. (2019). High-density ECoG improves the detection of high frequency oscillations that predict seizure outcome. *Clinical Neurophysiology*.

[8] Xie X., Pan X., Zhang W., An J. (2022). A context hierarchical integrated network for medical image segmentation. *Computers & Electrical Engineering*.

[9] Adorno Febles V. R., Blacksburg S., Haas J. A., Wise D. R. (2020). Translating the immunobiology of SBRT to novel therapeutic combinations for advanced prostate cancer. *Frontiers Oncology*.

[10] Peng J., Lalani A. K., Swaminath A. (2021). Cytoreductive stereotactic body radiotherapy (SBRT) and combination SBRT with immune checkpoint inhibitors in metastatic renal cell carcinoma. *Can Urol Assoc J*.

[11] Besson N., Pernin V., Zefkili S., Kirova Y. M. (2016). Evolution of radiation techniques in the treatment of mediastinal lymphoma: from 3D conformal radiotherapy (3DCRT) to intensity-modulated RT (IMRT) using helical tomotherapy (HT): a single-centre experience and review of the literature. *British Journal of Radiology*.

[12] Chen S. N., Ramachandran P., Deb P. (2020). Dosimetric comparative study of 3DCRT, IMRT, VMAT, Ecomp, and Hybrid techniques for breast radiation therapy. *Radiation Oncology Journal*.

[13] Solanki A., Kombathula S. H., Manna S. (2020). Adjuvant irradiation in carcinoma breast patients: comparison of 3DCRT and semi-automated complex VMAT hypofractionated plans. *Gulf Journal of Oncology*.

[14] Fields E. C., Rabinovitch R., Ryan N. E., Miften M., Westerly D. C. (2013). A detailed evaluation of TomoDirect 3DCRT planning for whole-breast radiation therapy. *Medical Dosimetry*.

